# Effect of Acute Static Stretching on the Activation Patterns Using High-Density Surface Electromyography of the Gastrocnemius Muscle during Ramp-Up Task

**DOI:** 10.3390/s21144841

**Published:** 2021-07-15

**Authors:** Noriaki Maeda, Makoto Komiya, Yuichi Nishikawa, Masanori Morikawa, Shogo Tsutsumi, Tsubasa Tashiro, Kazuki Fukui, Hiroaki Kimura, Yukio Urabe

**Affiliations:** 1 Department of Sports Rehabilitation, Graduate School of Biomedical and Health Sciences, Hiroshima University, 1-2-3 Kasumi, Minami-ku, Hiroshima 734-8553, Japan; makoto-komiya@hiroshima-u.ac.jp (M.K.); m-masanori@hiroshima-u.ac.jp (M.M.); shogo-tutumi@hiroshima-u.ac.jp (S.T.); tsubasatashiro716@hiroshima-u.ac.jp (T.T.); kazuki-fukui@hiroshima-u.ac.jp (K.F.); yurabe@hiroshima-u.ac.jp (Y.U.); 2 Faculty of Frontier Engineering, Institute of Science & Engineering, Kanazawa University, Kanazawa 920-1192, Japan; yuichi@se.kanazawa-u.ac.jp; 3 Department of Preventive Gerontology, Center for Gerontology and Social Science, National Center for Geriatrics and Gerontology, 7-430 Morioka-cho, Aichi, Obu City 474-8511, Japan; 4 Department of Rehabilitation, Hiroshima University Hospital, Hiroshima University, Hiroshima 734-8551, Japan; luna@hiroshima-u.ac.jp

**Keywords:** static stretching, motor unit recruitment, submaximal voluntary ramp contraction, high-density spatial electromyography

## Abstract

This study aimed to evaluate motor unit recruitment during submaximal voluntary ramp contraction in the medial head of the gastrocnemius muscle (MG) by high-density spatial electromyography (SEMG) before and after static stretching (SS) in healthy young adults. SS for gastrocnemius was performed in 15 healthy participants for 2 min. Normalized peak torque by bodyweight of the plantar flexor, muscle activity at peak torque, and muscle activation patterns during ramp-up task were evaluated before and after SS. Motor unit recruitment during the submaximal voluntary contraction of the MG was measured using SEMG when performing submaximal ramp contractions during isometric ankle plantar flexion from 30 to 80% of the maximum voluntary contraction (MVC). To evaluate the changes in the potential distribution of SEMG, the root mean square (RMS), modified entropy, and coefficient of variation (CV) were calculated from the dense surface EMG data when 10% of the MVC force was applied. Muscle activation patterns during the 30 to 80% of MVC submaximal voluntary contraction tasks were significantly changed from 50 to 70% of MVC after SS when compared to before. The variations in motor unit recruitment after SS indicate diverse motor unit recruitments and inhomogeneous muscle activities, which may adversely affect the performance of sports activities.

## 1. Introduction

Stretching is performed during warm-up for sports and is considered essential for optimizing performance [1,2]. It has proven to be an effective method for decreasing muscle stiffness [3], and improving joint flexibility and physical performance in activities involving jumping and dynamic balance [4]. In addition, stretching is important for the prevention of injuries during sporting activities [5]. There are several types of stretching, including static, dynamic, cyclic, and proprioceptive neuromuscular facilitation stretching; it is necessary to take into account the different effects of each stretching technique in order to identify one that would be best suited to the individual [3,4].

Static stretching (SS), which keeps the muscles stretched for a certain period of time without recoil to improve muscle flexibility, is widely used in the fields of sports and rehabilitation [3]. Current research suggests that SS decreases muscle activity and power and evokes contractile properties, thereby impacting physical performance [6]. A previous study reported greater activation deficits after SS than before SS [7]. These effects may have been due to peripheral nerve mechanisms resulting from muscle tendon stiffness [8]. To date, a unified view on the effects of SS is lacking.

Multichannel spatial electromyography (SEMG) was recently developed as a noninvasive technique to measure the mechanical and contractile properties of the motor unit activity of skeletal muscles using multiple electrodes arranged on a two-dimensional plane [9]. SEMG measures the number of motor units recruited by an individual during active muscle contraction [10,11,12]. Holtermann et al. [13] suggested that changes in the spatial distribution of SEMG could be explained by physiological changes in motor unit recruitment. These changes are underpinned by the high density of muscle fibers in a limited area and the spatial inhomogeneity of muscle fibers for certain muscles [14].

Previous studies have demonstrated that the muscle contractions induced by electrical stimulation to the muscle belly are prolonged after performing SS and induce a decrease in muscle and tendon stiffness [15]. However, to our knowledge, there is no previous study about the differences in motor unit recruitment during submaximal voluntary ramp contraction before and after SS being unclear. Therefore, this study aimed to evaluate motor unit recruitment during submaximal voluntary ramp contraction in the medial head of the gastrocnemius muscle (MG) by SEMG performed after SS compared to before SS in healthy young adults. We hypothesized that variations in motor unit recruitment would indicate diverse motor unit recruitments during ramped submaximal voluntary contraction performed after SS when compared to that before SS.

## 2. Materials and Methods

### 2.1. Experimental Design

In this study, a randomized repeated measures experimental method was used to examine the effects of SS on plantar flexion torque and MG activation patterns by performing isometric plantar flexion exercises in the range of 30 to 80% of the maximal voluntary contraction (MVC). Stretching was performed on the dominant limb, to be assessed unilaterally, in order to ensure consistency in data collection across subjects. The dominant hand was defined as the limb used to kick the soccer ball. All participants were determined to be right-leg dominant [16].

**Figure 1:**
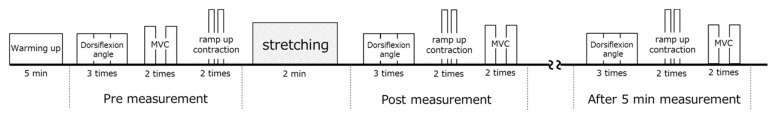
Flowchart of the measurement procedure. MVC: maximal voluntary contraction.

The procedure is shown in Figure 1. The warm-up consisted of a 5-min cycling exercise with the ergometer air resistance set to 75 W and the cadence set to 60 rpm, followed by a complete break for 3 min before the start of the test [17]. We then measured the normalized peak torque (NPT) using a Biodex 4 dynamometer (Sakai Medical Co., Ltd., Tokyo, Japan) and muscle activation patterns. The activation patterns of the MG were measured using SEMG when performing ramped submaximal contractions during isometric ankle plantar flexion from 30 to 80% of MVC before and immediately after SS or at 5 min after SS. Stretching was performed on the dominant leg using the Biodex 4 dynamometer in a seated position. The foot was placed at maximum dorsiflexion on the footplate using a stretching device with the Biodex 4 dynamometer while the knee joint was placed in the neutral position. The stretch was maintained for 2 min based on a former report by Kanazawa et al. [18], which stated that maximum dorsiflexion reached a plateau at 2 min when both the gastrocnemius and Achilles tendon underwent SS at maximum dorsiflexion in a sitting position. The degree of passive dorsiflexion was recorded using the Biodex 4 dynamometer. The maximum degree of ankle dorsiflexion was defined as the angle at which the subjects began to experience pain.

### 2.2. Participants

In total, 16 healthy male recreationally active subjects (mean age ± standard deviation [SD], 24.7 ± 2.4 years; height, 170.7 ± 6.1 cm; body mass, 63.2 ± 8.2 kg) voluntarily participated in this study. “Recreationally active” was defined as participating in at least one exercise session per week in the past two months and not participating in any structured exercise training during this period. We excluded participants with injuries in the lower extremities and/or neuromuscular disease. Subjects performed both conditions in various orders in a two-day period with intervals of no less than 24 h and no more than 48 h between tests. All of the subjects were able to complete the task. Three successful trials were conducted in each condition and were used for data analysis. The average duration of trials in each laboratory was 50.0 ± 4.0 min.

All subjects provided informed consent to participate in the study. The study protocol met the requirements of the Declaration of Helsinki and was approved by the Ethical Committee for Epidemiology of Hiroshima University (approval number E-2006).

### 2.3. Assessment of Normalized Peak Torque of Plantar Flexor

In order to assess the peak torque of the plantar flexor muscle, participants were instructed to plantar flex their ankle gradually by maximally contracting the muscle for 0 to 5 s and maintaining it for 2 of the 5 s. Subjects performed at least two MVC trials with >120 s rest between trials after one or two practice sessions [19].

### 2.4. Assessment of SEMG

After NPT, subjects were instructed to perform a forceful ramp contraction from 0 to 80% of MVC with a rate of increase of approximately 10% of the MVC per second [20]. Subjects were presented with the target torque generated on the monitor of a personal computer and practiced performing MVC and ramp submaximal contractions for >10 min before the session.

### 2.5. SEMG Recording

**Figure 2:**
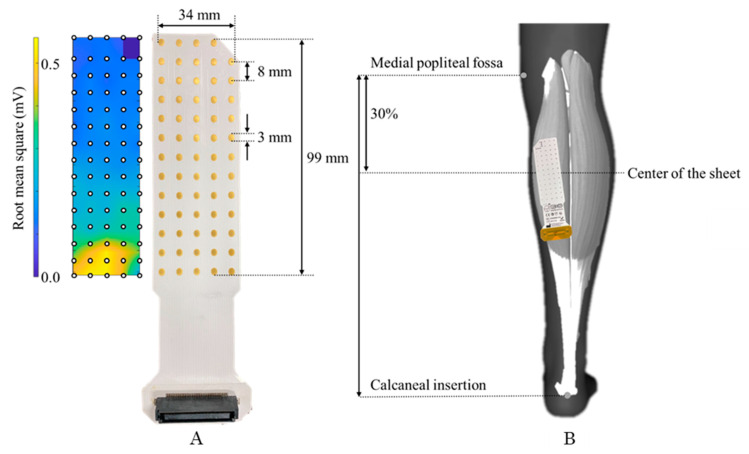
The location of surface electromyography application and amplitude values of electrodes. (**A**): A color map of the typical spatial distribution pattern of the amplitude values of a surface electromyogram. The grid consists of 13 rows and five columns. In the present study, one site of the proximal part of the MG muscle (upper right part of the electrode) was missing. (**B**): The electrode grid of the multichannel electromyography was placed at the medial head of the gastrocnemius muscle. The central part of the electrode grid was attached to the center of the line connecting the medial knee fossa and calcaneal insertion. Reference electrodes were attached at the position of the fibular head and patella.

The SEMG signals of MG were recorded using a 64-electrode semi-disposable grid (ELSCH064RS3, OT Bioelettronica, Torino, Italy) using the same procedure as that used in previous studies [20]. The grid consisted of five rows and 13 columns of electrodes (1-mm diameter, 8-mm distance between electrodes), with one missing electrode in the upper left corner (Figure 2). Before attaching the electrodes to the skin with an adhesive sheet (KITAD064, OT Bioelettronica), the calf hair was removed, the skin was cleaned with alcohol, and a conductive paste (Elefix Z-181BE, NIHON KOHDEN, Tokyo, Japan) corresponding to the electrode placement was applied.

The SEMG electrode grid was placed on the MG. The center of the electrode grid was attached to the MG near the point of the maximal cross-sectional area of the lower leg, which was at a level of 30% of the lower leg length from the medial popliteal fossa to the calcaneal insertion (Figure 2). Before attaching the electrode sheet, ultrasound measurements were performed underneath to confirm whether the MG was included in the area of the electrode sheet. The rows of the electrode grid were placed parallel to the longitudinal axis of the MG, and the site of the missing electrode was proximal to the MG based on a previous study [20]. Thereafter, the electrode grid was attached to the computer through a 12-bit analog-to-digital converter (EMG-USB2+, OT Bioelettronica). The recording of the SEMG signals was controlled using a software program (OTBioLab+ v1.4.2.0, Bioelettronica, Torino, Italy). Two reference electrodes were attached at the fibular head and the center of the patella, respectively. The same investigator performed all procedures. The SEMG monopole signal was amplified by a factor of 1000, sampled at 2048 Hz per channel, and converted to digital data using a 12-bit analog-to-digital converter. The recorded monopolar signals were bandpass-filtered (10–500 Hz) off-line and analyzed using software (MATLAB 2018a, MathWorks GK, Massachusetts, USA). Bipolar multichannel SEMG signals (*n* = 59) along the column were segmented from 64 electrodes; SEMG signals were sampled between 3 s (30% of MVC) and 8 s (80% of MVC) of the ramp-up protocol, and the root mean square (RMS) was calculated. The RMS estimates were normalized by the value of MVC; the coefficient of variation (CV) of force (SD/mean x100, CV force) was calculated in the same time series as the coefficient of variation of SEMG variables [20]. Modified Entropy, RMS, and CV were determined to characterize the heterogeneity of the potential distribution of SEMG at each contraction time. The modified entropy was obtained over a 1 s epoch obtained during a tilted submaximal contraction, and this value was calculated by the following equation after calculating 59 RMS values (in space) of a single difference signal from the EMG amplitudes of muscle activity in a wide range of muscles [11]:

$$E=\;-\;\overset{59}{\underset{i\;=\;1}{\sum }}p{\left(i\right)}^{2}\log_{2}p{\left(i\right)}^{2}$$

The p(i) in the above equation is the square of the RMS value of channel i divided by the sum of the squares of the 59 RMS values at a given contraction time. Thus, the normalized power of each channel was expressed as p(i)^2^. The CV of RMS was defined as the mean value of the 59 RMS measurements at each time point and a quarter of the SD of the 59 RMS measurements. We calculated the change in two variables (modified entropy and CV of RMS) to examine the change in the recruitment of motor units over time. These variables were calculated between contraction times of 2 s and 8 s. The decrease in the modified entropy and the increase in the CV of RMS indicated an increase in the heterogeneity of the SEMG potential distribution in the electrode grid [21]. Recent studies have quantified the spatial distribution pattern of SEMG and used SEMG to estimate the recruitment pattern of motor units [22].

### 2.6. Statistical Analysis

Data were analyzed using EZR (Saitama Medical Centre, Jichi Medical University, Saitama, Japan) [23]. Continuous data are presented as the mean ± SD or median (min, max). Before the analysis, data normality was confirmed using the Shapiro–Wilk test. A one-factor repeated measure ANOVA was conducted to compare NPT, RMS, modified entropy, and CV among the conditions (pre, post, and post-5 min) if the normality was confirmed. A Friedman test was conducted if the abnormality was confirmed for each condition (pre, post, and post-5 min). Differences between torque levels were analyzed using the Holm post hoc test to adjust the p value for multiple comparisons. A significance level of *p* < 0.05 was used.

## 3. Results

**Figure 3:**
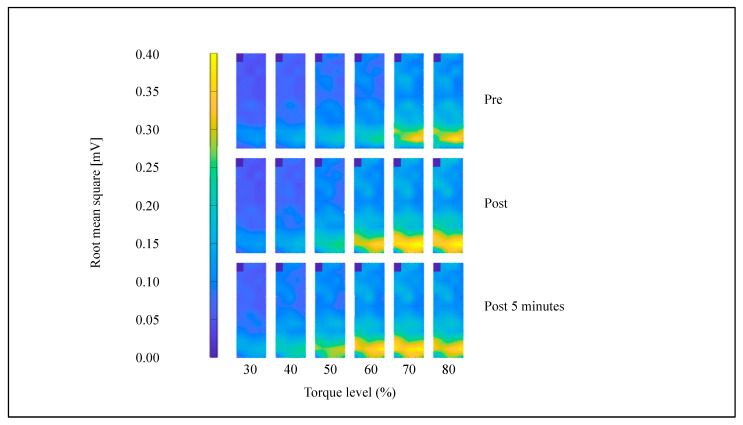
Root mean square of multichannel surface electromyogram amplitudes at 30 to 80% of the maximum voluntary contraction between conditions.

**Figure 4:**
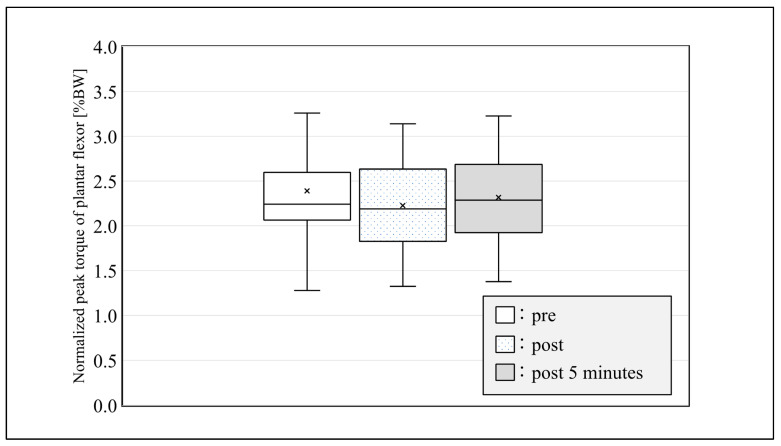
Normalized peak torque of the plantar flexor at pre, post, and post-5-min conditions.

The color map of representative multichannel SEMG amplitudes is shown in Figure 3. Differences in the potential distribution of SEMG at each contraction time pre- and post-SS were observed. Significant differences were not noted between NPT immediately after SS and NPT at 5 min after SS (Figure 4).

**Figure 5:**
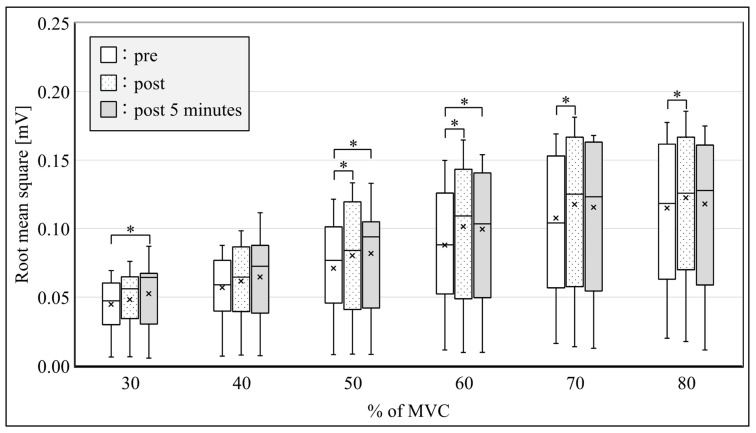
Root mean square of multichannel surface electromyogram amplitudes during ramp contractions at pre, post, and post-5-min conditions. * *p* < 0.05. MVC: maximal voluntary contraction.

**Figure 6:**
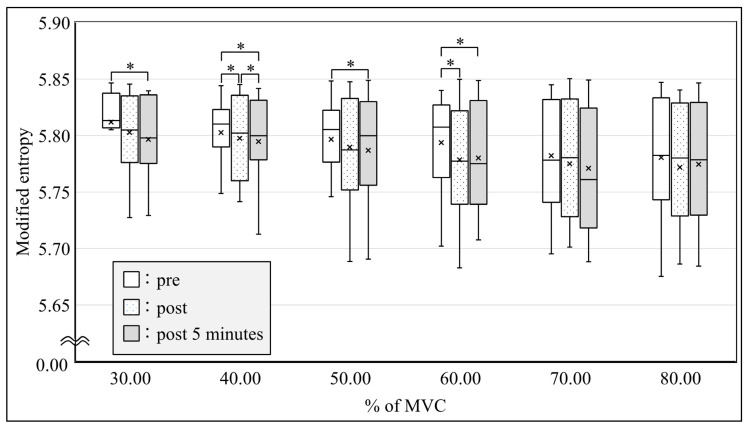
Comparison of the modified entropy in the amplitude of the multichannel surface electromyography during ramp contractions between pre, post, and post-5-min conditions. * *p* < 0.05. MVC: maximal voluntary contraction.

**Figure 7:**
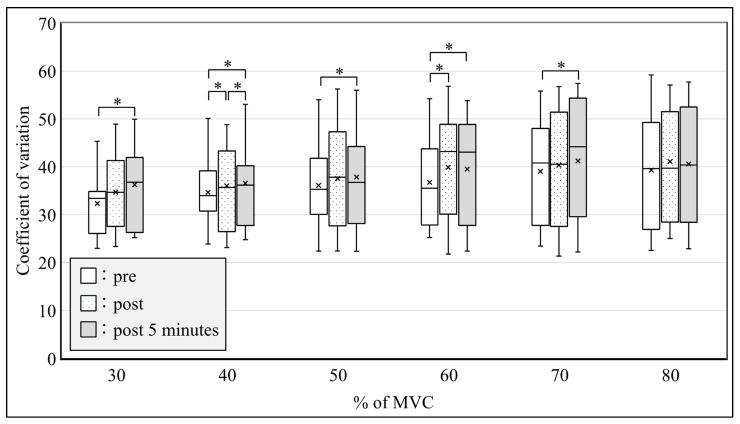
Coefficient of variation in the amplitude of the multichannel surface electromyography during ramp contractions at pre, post, and post-5-min conditions. * *p* < 0.05. MVC: maximal voluntary contraction.

All subjects were able to perform the submaximal ramp contraction from 10 to 80% of MVC. RMS after SS was significantly increased from 50 to 80% of MVC immediately after SS and from 50 to 60% of MVC at 5 min after SS (Figure 5, *p* < 0.05) in the MG. Significant differences were observed in the modified entropy and CV between pre-, immediately post-, and 5-min post-SS. The modified entropy of RMS from 40 to 60% of MVC immediately after SS and from 30 to 60% of MVC at 5 min after SS was significantly lower than that before SS (Figure 6, *p* < 0.05). In contrast, the CV of RMS from 30 to 60% of MVC immediately after SS and from 30 to 70% of MVC at 5 min after SS was significantly higher than that before SS (Figure 7, *p* < 0.05).

## 4. Discussion

This study evaluated, in healthy young adults, the motor unit recruitment during submaximal voluntary ramp contraction in MG by an SEMG performed after SS compared to one performed before it. The primary results of the present study were as follows: the acute effects on the motor unit recruitment during ramped submaximal voluntary contraction after SS exhibited (1) significantly lower NPT and (2) significant changes in RMS, modified entropy, and CV after SS compared to before SS. These findings supported our hypothesis that variations in motor unit recruitment indicated diverse motor unit recruitments during ramped submaximal voluntary contractions performed after SS relative to those performed before it.

NPT of the plantar flexors was significantly lower immediately after and at 5 min after SS when compared to before it. Studies have reported decreased muscle power or evoked contractile properties after SS in the acute phase [9,24,25,26], which is in accordance with our findings. For these reasons, peripheral nerve mechanisms (more specifically, reduced muscle tendon stiffness) caused muscle weakness immediately after SS [26,27,28,29,30]. However, Magunusson et al. [31] reported that continued stretching of the muscle-tendon complex decreased the static torque. Therefore, it is necessary to analyze whether the type, intensity, frequency, and duration of stretching of the muscle-tendon complex reduces muscle exertion.

All subjects completed the ramp-up contraction task. The RMS of 50 to 80% of MVC immediately after SS and the RMS of 50 to 60% of MVC at 5 min after SS exhibited a greater force fluctuation in the ramp-up contraction task than that before SS. In this study, the muscle force fluctuation after SS required a significantly greater activation of MG than that before SS, which may have influenced the accuracy of the muscle force before SS to match the required force level.

These results indicated that the motor unit recruitment immediately after SS and at 5 min after SS involved diverse motor unit recruitments and inhomogeneous muscle activation patterns during the contraction task compared to that before SS. Watanabe et al. [21] reported that the values of modified entropy and CV indicated the heterogeneity of the spatial multichannel SEMG potential distribution within the electrode grid. Spatially distinct and changing functional properties are expected during muscle contraction, and spatially nonuniform SEMG signals are likely to be noted within the individual muscles [32]. Moreover, Farina et al. [11] suggested that the heterogeneity during a static contraction was related to the endurance time of muscles and was in line with the effect of SS for muscle conditioning. The potential for changes in the SEMG distribution during ramped submaximal contraction after SS may be related to peripheral changes, such as size changes in the motor unit area, location, type, distribution, and central nervous system adaptation. Generally, motor units are recruited to exert less force to enable muscles to contract at lower forces, and larger muscles are recruited when higher forces are required with ramp-up contraction tasks. Abnormalities may arise from reductions in musculotendinous stiffness [8,27,29]. Although our findings may only partially encapsulate the physiological changes after SS, they provide pertinent insights into the changes in the motor unit recruitment and inhomogeneous muscle activation patterns during ramped submaximal contraction performed after SS. SS can cause sagging by inducing stretching of the musculotendinous unit (MTU), resulting in increased electromechanical delays that affect force transfer and may reduce elastic energy [33]. In addition, Power et al. [34] also found that SS increased tendon compliance, thereby reducing force production.

Several limitations of the study need to be considered. The SEMG amplitude estimates combine properties in the peripheral and central motor units and only provide a crude estimate of motor unit recruitment [35]. Further research that utilizes analytical methods is needed to elucidate the mechanisms underscoring the inhomogeneous muscle activation patterns before and after SS. Second, we only assessed MVC and muscle activation patterns during ramped submaximal voluntary contractions, but not those of physical performances such as jump height. Further studies are needed to investigate the effects after SS on other muscle activation patterns and physical performance. Third, the possibility of changes in the SEMG distribution during tilted submaximal contraction after SS is thought to be related to peripheral changes, such as size changes in the area, location, type, and distribution of motor units and adaptations of the central nervous system, so this is unclear until the same changes occur in muscles other than MG.

## 5. Conclusions

In conclusion, our findings demonstrate that variations in motor unit recruitment indicate diverse motor unit recruitments and inhomogeneous muscle activation patterns during ramped submaximal contractions performed after SS. Moreover, SS can negatively affect the muscle activation patterns during submaximal ramp contractions that are performed after it, which may affect movement during sports activities after SS. Further research is needed in order to understand the underlying mechanisms.

## Data Availability

Not applicable.
